# Evaluation of the Glymphatic System With Diffusion Tensor Imaging-Along the Perivascular Space in Cancer Pain

**DOI:** 10.3389/fnins.2022.823701

**Published:** 2022-03-04

**Authors:** Aibo Wang, Lei Chen, Can Tian, Xiaoyu Yin, Xinyue Wang, Yize Zhao, Miao Zhang, Lili Yang, Zhaoxiang Ye

**Affiliations:** ^1^Department of Radiology, Tianjin Medical University Cancer Institute and Hospital, Tianjin, China; ^2^National Clinical Research Center for Cancer, Tianjin, China; ^3^Tianjin’s Clinical Research Center for Cancer, Tianjin, China; ^4^The Key Laboratory of Cancer Prevention and Therapy, Tianjin, China; ^5^Department of Cancer Pain Management, Tianjin Medical University Cancer Institute and Hospital, Tianjin, China; ^6^Department of Pathology, Tianjin Third Central Hospital, Tianjin, China; ^7^College of Medical Imaging, Dalian Medical University, Dalian, China; ^8^Zhejiang MedicalTech Therapeutics Company Co., Ltd., Wenzhou, China

**Keywords:** cancer pain, glymphatic system, MRI, DTI, ALPS

## Abstract

Cancer pain (CP) is one of the most common symptoms affecting life quality, and there is considerable variation in pain experience among patients with malignant tumors. Previously, it has been found that the fluid drainage function in the brain can be regulated by peripheral pain stimulation. However, the relationship between cancer pain and functional changes of the glymphatic system (an important pathway for fluid drainage in the brain) remains unclear. In this study, 97 participants were enrolled, which included 40 participants in the cancer pain (CP) group, 27 participants in the painless cancer (PLC) group and 30 participants in the control (NC) group. Differences in glymphatic system function among the three groups and between before and after pain pharmacological intervention were analyzed by measuring diffusivity and the index along the perivascular space (ALPS index) using diffusion tensor imaging. We found that diffusivity and the ALPS index were significantly lower in the CP group than in the PLC and NC group and increased following intervention with pain relief. Moreover, the ALPS index was negatively correlated with the degree of pain in the CP group. The present study verified that alterations in glymphatic function are closely related to cancer pain, and the quantification of functional changes reflects pain severity. Our findings support the use of neuroimaging biomarkers for cancer pain assessment and indicate that pain can be alleviated by regulating brain function status.

## Introduction

Cancer pain (CP) is a general term for pain caused by tissue and nerve invasion due to a primary tumor or metastasis as well as pain caused by tumor-related treatment and is one of the most common symptoms in cancer patients, and incidence of pain accounts for 40–70% of all cancer patients (Neufeld et al., 2017; Yang et al., 2021). CP not only causes significant physical and psychological pain to patients, impacting quality of life, but also presents difficulties in cancer treatment (Ham et al., 2017). Thus, active and effective control of CP and improving life quality of cancer patients through the accurate assessment of the pain are considered important tasks during cancer treatment. The mechanism of CP is complex, and pain perception varies considerably among cancer patients (Loffler et al., 2018; Caraceni and Shkodra, 2019). Currently, clinical assessment of the degree of CP is primarily based on a patient’s subjective pain score, dose of pain medication, and observation of status by physicians (Jeter et al., 2018; Caraceni and Shkodra, 2019). However, patients’ subjective cognition and expression ability and doctors’ subjective misjudgment may result in the pain assessment to deviate from actual pain status, which may lead to inaccurate drug dosages, delayed pain control (Neufeld et al., 2017; Sato et al., 2017), unsatisfactory pain relief, or even drug overuse during CP interventions (Preuss et al., 2021). Therefore, the objective quantification of CP will help in the formulation of effective pain intervention plans and adjustment of treatment plans based on corresponding changes in indicators caused by pain.

The glymphatic system is a recently discovered effective drainage and exchange pathway between the cerebrospinal fluid (CSF) in subarachnoid space and the interstitial fluid (ISF) in the brain parenchyma. It is composed of the perivascular space and the water channel aquaporin-4 (AQP4), localized on the endfeet of astrocytes (Iliff et al., 2012; Nycz and Mandera, 2021), and is active during deep sleep (Anzai and Minoshima, 2021). The glymphatic system, the extracellular space (ECS) in the deep brain, the subarachnoid space, the lymphatic vessels in the dural sinuses, and the ventricular system together constitute the intracerebral fluid circulation pathway (Louveau et al., 2015; Lei et al., 2017; Nycz and Mandera, 2021). Fluid drainage in the brain is crucial for maintaining the homeostasis of the brain microenvironment (Lei et al., 2017). Increasingly, studies have shown that impairment of the drainage and clearance function of the glymphatic system is closely related to several mental and neurodegenerative diseases, such as depression, Alzheimer’s disease (AD), and Parkinson’s disease (PD) (Lei et al., 2017; Rasmussen et al., 2018; Hablitz and Nedergaard, 2021; Yan et al., 2021). Furthermore, several studies have reported that peripheral pain stimulation induces spatial structural alterations and decreased drainage function of the ECS in the deep brain (Goldman et al., 2020; Li et al., 2020). More importantly, chronic pain is a vital risk factor for depression and cognitive impairment (Jenny Wei et al., 2021). Therefore, CP may also cause alterations in glymphatic system function. However, whether glymphatic system function is altered in patients with CP and whether pain and glymphatic system function are correlated remain unclear. This knowledge will help gain a deeper understanding of brain changes under CP and whether changes in brain functional parameters have potential value for the quantitative evaluation of CP.

**FIGURE 1:**
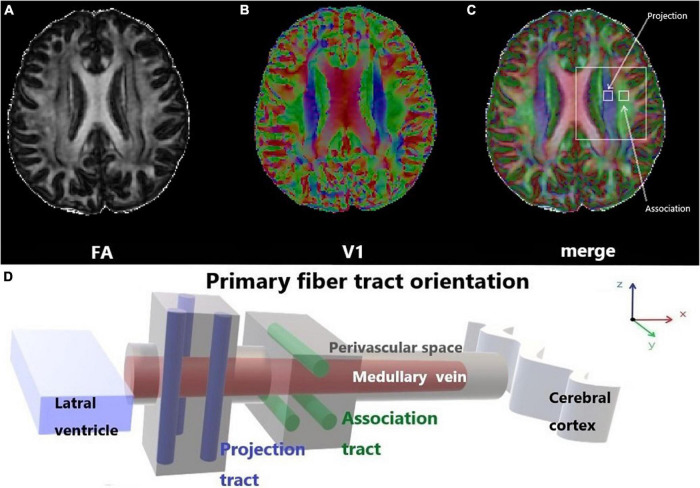
Diffusion tensor imaging-along the perivascular space (DTI-ALPS). **(A)** The DTI fractional anisotraphy (FA) map shows the maximum level of the lateral ventricle for anatomical location. **(B)** The DTI V1 map shows the direction and distribution of the different types of fiber tracts: projection fibers (*z*-axis: blue), association fibers (*y*-axis: green), and subcortical fibers (*x*-axis: red). **(C)** The merged image of the FA and V1 maps and two regions of interest (ROIs) were set onto the projection and association fibers, and diffusivity within the ROIs in the three directions was measured. **(D)** Schematic of the positional relationship between the perivascular space of the medullary vein and adjacent fibers at the same level. The perivascular space of the medullary vein is parallel to the *x*-axis and orthogonal to the projection fibers in the *z*-axis and the association fibers in the *y*-axis.

Previous studies on the glymphatic system have been based primarily on the observation of tracer drainage, diffusion, and distribution following invasive tracer introduction (Lei et al., 2017; Benveniste et al., 2021). A non-invasive method to assess glymphatic system function will offer significant value in clinical settings. Diffusion tensor imaging (DTI)-along the perivascular space (DTI-ALPS) was proposed by Taoka et al. (2017) as a non-invasive measurement method, which is now widely used in studies on the glymphatic system of the human brain. This method assumes the following: in the white matter near the lateral ventricles, the medullary veins run in the right-left direction (x-axis), which is perpendicular to the lateral ventricular wall. In contrast, at this level, the projection fibers run predominantly in the head-foot direction (*z*-axis), mainly adjacent to the lateral ventricle, and the association fibers (the superior longitudinal fascicles) run in the anterior-posterior direction (*y*-axis), outside of the projection fibers (Taoka et al., 2017). Thus, the perivascular space is orthogonal to the major fibers at this level (Taoka et al., 2017). In our previous study, we found that myelinated fibers in the deep brain play a crucial role in regulating ISF drainage (Wang et al., 2019). The application of a high b-value (e.g., *b* = 1000 s/mm^2^) to DTI suppresses the flowing venous blood, which allows independent analysis of the diffusivity along the x-direction in perivascular space (Taoka et al., 2017; Figure 1).

By DTI-ALPS, Taoka et al. (2017) found that the ALPS index was significantly negatively correlated with the Mini-Mental State Exam score in AD patients when b = 1000 s/mm^2^, which indicated lower water diffusivity along the perivascular space in relation to AD severity. One study in 2020 on glymphatic system alterations in patients with type two diabetes showed that diffusivity in different fiber areas and the ALPS index were associated with the severity of diabetes; moreover, the ALPS index reflected the damage of the glymphatic system (Yang et al., 2020). Recently, McKnight et al. (2021) reported that the ALPS index in PD patients was significantly lower than that in patients with essential tremor, which may be related to changes in the transport environment in the glymphatic system due to abnormal protein aggregation in PD. Furthermore, they found correlations between the ALPS index and age and T2-weighted white matter hyperintensity (McKnight et al., 2021). Taken together these findings demonstrate that DTI-ALPS is feasible in detecting functional changes of the glymphatic system. In the present study, we applied DTI-ALPS to the analysis of ALPS index changes in participants experiencing CP and investigated the relationship between the ALPS index and CP to determine the potential value of the ALPS index as a biological indicator of CP conditions.

## Materials and Methods

### Informed Consent, Participant Recruitment, and Criterion

The study was approved by the ethics committee of Tianjin Medical University Cancer Institute and Hospital, and all participants provided informed consent, adhering to the ethical standards stipulated by the Declaration of Helsinki and its amendments.

**FIGURE 2:**
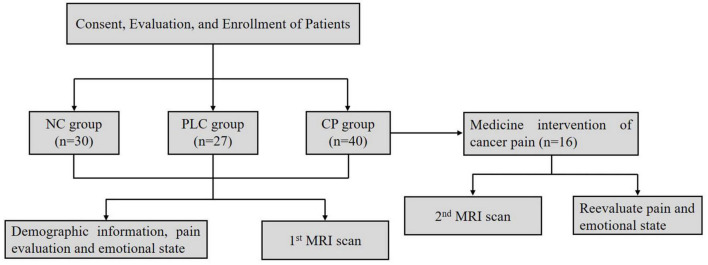
Procedures and data used for the study.

Ninety seven participants were enrolled, which included Forty participants in the CP group, Twenty seven participants in the PLC group and Thirty participants in the NC group (Table 1). All subjects were right-handed. After enrollment, we acquired brain magnetic resonance imaging (MRI) data, which included axial T2-fluid attenuated inversion recovery (FLAIR) images and DTI on a 3.0-Tesla magnetic resonance scanner (Discovery MR750, General Electric, Milwaukee, WI, United States) to evaluate T2-weighted hyperintensities in the deep white matter (DWM) and obtain ALPS indices. The self-rating anxiety (SAS) and self-rating depression scales (SDS) were used to evaluate the emotional states of the two groups. The numeric rating (NRS) and visual analog scales (VAS) were used to evaluate the degree of pain in the CP group. The mini-mental state examination (MMSE) was used to evaluate the cognitive function. Sixteen participants in the CP group were included in the follow-up group, after CP pharmacological intervention, according to the ‘three-stage’ treatment plan and treatment for primary tumors for 1 month without other treatments, such as nerve block and physiotherapy. Patients’ degree of pain, emotional state, and brain MRI were assessed using the same methods as those used at baseline. None of the 16 participants were on antidepressants or anti-anxiety drugs during the study period (Figure 2).

**TABLE 1:** Demographic and clinical information of each group.

	CP group (*n* = 40)	PLC group (*n* = 27)	NC group (*n* = 30)
Recruitment criteria	• confirmed pain related to spinal bone metastasis • pain duration ≥ 1 month • no obvious pain feeling in any other parts of the body • no acute symptoms of tumor in the last month • no invasive treatment in the last month • no cognitive impairment • no mental illness or serious consciousness disturbance • no drug abuse or alcohol addiction • no heart, liver or kidney failure • expected survival time > 2 months	• confirmed tumor history • no obvious pain feeling • no acute symptoms of tumor • no invasive treatment in the last month • no cognitive impairment • no mental illness or serious consciousness disturbance • no drug abuse or alcohol addiction • no heart, liver or kidney failure • expected survival time > 2 months	• healthy adult • no chronic pain • no chronic diseases • no invasive treatment in the last month • no anxiety or depression • no cognitive • impairment • no mental illness or serious consciousness disturbance • no drug abuse or alcohol addiction • no heart, liver or kidney failure • expected survival time > 2 months
Primary tumor	• lung cancer (19 cases) • breast cancer (14 cases) • prostatic carcinoma (3 cases) • renal carcinoma (2 cases) • rectum carcinoma (1 case) • thyroid carcinoma (1 case)	• lung cancer (19 cases) • breast cancer (8 cases)	
Primary tumor treatment	• systemic chemotherapy (15 participants) • targeted therapy (12 participants) • chemotherapy combined with targeted therapy (7 participants) • no treatment as first diagnosed (6 participants)	• systemic chemotherapy (10participants) • targeted therapy (6 participants) • chemotherapy combined with targeted therapy (8 participants) • no treatment as first diagnosed (3 participants)	
Pain intervention	• no pain treatment as first diagnosed (6 participants) • did not undergo pain intervention as focusing on primary tumor treatment (9 participants) • did not respond well to pain medication (25 participants)		
Age (years)	54.33 ± 7.28	56.30 ± 6.31	52.77 ± 10.34
Age composition			
≥ 65	4 (10%)	2 (8%)	6 (20%)
≥ 45, < 65	32 (80%)	23 (92%)	17 (56.67%)
≥ 30	4 (10%)	1 (4%)	7 (23.33%)
Sex			
Male	19 (47.5%)	12 (44.4%)	18 (60%)
Female	21 (53.5%)	15 (55.6%)	12 (40%)
SAS	58.98 ± 8.22	52.07 ± 4.66	44.80 ± 3.55
SDS	58.80 ± 7.79	56.77 ± 6.83	48.10 ± 2.66
MMSE	28.4 ± 1.21	29.4 ± 1.13	28.8 ± 1.17
Fazekas score			
0	14 (35%)	9 (33.3%)	9 (30%)
1	12 (30%)	9 (33.3%)	13 (43.3%)
2	12 (30%)	9 (33.3%)	7 (23.3%)
3	2 (5%)	0	1 (3.3%)

*SAS, self-report anxiety scale; SDS, self-report depression scale; MMSE, mini mental state examination.*

### Cancer Pain Group

Forty participants were recruited into the CP group, which included 19 males (47.5%) and 21 females (53.5%), with an average age of 54.33 ± 7.28 years. All participants in the CP group had a confirmed history of spinal bone metastasis, and primary tumors included lung cancer (19 cases), breast cancer (14 cases), prostatic carcinoma (3 cases), renal carcinoma (2 cases), rectum carcinoma (1 case), and thyroid carcinoma (1 case). All participants in the CP group have no obvious pain feeling in any other parts of the body except for the confirmed back pain related to spinal bone metastasis diagnosed via various imaging examinations, which included patients who had not taken any pain treatment medicine (15 participants) as some of these participants were diagnosed with tumor for the first time and some of these participants were focusing on treatment of the primary tumor by systemic chemotherapy or targeted therapy and did not yet undergo intervention targeting the pain as well as those who did not respond well to pain medication before enrollment (25 participants). Some participants showed the symptoms related to primary tumor such as cough, blood in the sputum, chest tightness, malnutrition and enlarged axillary lymph nodes, those did not affect the nervous system directly. Pain duration of each participant was ≥ 1 month. The average pain duration time before hospitalization was 6.46 ± 4.50 months. All participants in the CP group have no acute symptoms and no invasive treatment in the last month.

### Painless Cancer Group

Twenty seven participants were recruited into the PLC group, which included 19 males (44.4%) and 21 females (55.6%), with an average age of 56.30 ± 6.31 years. All participants in the PLC group had a confirmed history of tumor including lung cancer (19 cases), breast cancer (8 cases). All participants in the PLC group have no obvious pain feeling in any parts of the body except for the symptoms related to primary tumor such as cough, blood in the sputum, chest tightness, malnutrition and enlarged axillary lymph nodes, those did not affect the nervous system directly. Except for three participants whose tumors were first diagnosed, the rest of the participants in the PLC were receiving systematic chemotherapy or targeted therapy for tumors. All participants in the PLC group have no acute symptoms and no invasive treatment in the last month.

### Normal Control Group

Thirty participants were recruited into the control (NC) group, which included 18 males (60%) and 12 females (40%), with an average age of 52.78 ± 10.34 years. All participants in the NC group were healthy people without chronic pain or chronic diseases or anxiety or depression in the past and have no acute symptoms and no invasive treatment in the last month.

### Assessment of Pain Level

The NRS and VAS were used to quantify the degree of pain in the CP group. The NRS is an 11-point scale from 0 to 10. A higher number indicates greater pain: 0 indicates “no pain,” and 10 indicates the “worst imaginable pain.” Participants selected a number that best represented their pain. The VAS is a reliable, valid, responsive, and frequently used scale to measure pain outcomes. It consists of a bidirectional straight 10-cm line with two labels at each end of the line: “no pain” and “worst possible pain.” Patients were instructed to draw a vertical mark on the line that indicated their pain level (Hjermstad et al., 2011).

### Emotional State Evaluation

The SDS and SAS were used to evaluate the emotional states of the NC and CP groups. The SDS is a self-report instrument designed to detect symptoms related to depression and measure the severity of depression. The SAS is designed to detect symptoms related to anxiety. The two scales are similar in the items and specific methods in scale assessment. Both the SDS and SAS comprise 20 items, each of which is scored on a scale of 1–4, ranging from the absence of the symptom (score of 1) to maximal symptoms (score of 4), and a higher score indicates greater severity of depression or anxiety. Standard scores of 53 (equal to the original raw score of 41) for the SAS and 50 (equal to the original raw score of 40) for the SDS were used as the cut-off scores for Chinese clinical significance, where the higher the score, the more severe the depressive or anxious mood (Zhang et al., 2021).

### Cognitive Evaluation

The MMSE is a screening instrument to acquire a global impression of cognitive function. It was administered as the first instrument in a comprehensive fitness-to-drive assessment in a clinical setting (see Piersma et al. (2016) for the full protocol). The sum score of the MMSE (range 0–30) was used.

### Brain Magnetic Resonance Imaging

#### Image Acquisition

For the axial T2-FLAIR image, we used the following scanning protocols: repetition time [TR]/echo time [TE] = 8000/120 ms; inversion time [TI] = 2100 ms; flip angle = 90°; section thickness = 5 mm; field of view [FOV] = 26; number of excitations = 1. The scanning protocols for the DTI were: b = 0 and b = 1000 s/mm^2^, echo-planer sequence, TR = 6600 ms, TE = 89 ms, motion probing gradient (MPG) = 60 directions, FOV = 230 mm, matrix = 94 × 94, slice thickness = 3 mm.

#### Image Analysis

##### White Matter Lesion Assessment

Brain axial T2-FLAIR images were assessed by two radiologists with more than 5 years of diagnostic experience. DWM T2-weighted hyperintensity was graded on a 0–3-point scale according to the Fazekas score standard: absence (score of 0); punctate lesions (score of 1); punctate lesions beginning to merge (score of 2); large lesion fusion (score of 3) (Morotti et al., 2020).

##### Diffusion Tensor Imaging Analysis and Along the Perivascular Space Calculation

**FIGURE 3:**
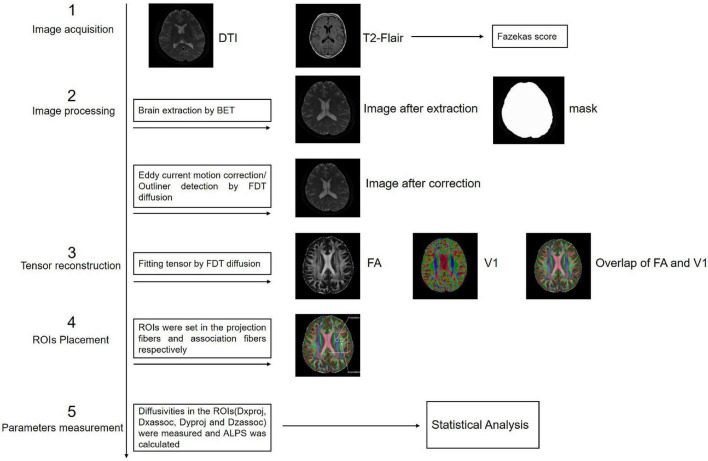
Process of the DTI analysis and ALPS index calculation.

The FMRIB Software Library (FSL) toolkit (version 6.0)^1^ was used to process and reconstruct the DTI images. First, the BET toolkit (version 2.0) was used to extract the brain, and the fractional intensity threshold was set to 0.3. Images were then motion-corrected and eddy current-corrected using the eddy function in the FDT diffusion toolkit (version 2.0). The processed DTI images were used to fit the tensor using the FDT diffusion toolkit (version 2.0), and the parameter images were generated to allow measurements of FA, tensor, diffusivity, and vector. The locations of projection and association fibers adjacent to the left lateral ventricular were confirmed by two radiologists with more than 5 years of diagnostic experience. Two 3 mm × 3 mm regions of interest (ROIs) were set in each of the two fibers in FSLeyes. Diffusivity of the three directions in the ROIs was measured and the mean ALPS index was calculated according to the following formula:

Along the perivascular space index = mean (Dxproj, Dxassoc)/mean (Dyproj, Dzassoc) (1),

where Dxproj and Dxassoc are the *x*-axis diffusivities in the area of the projection and association fibers, respectively, Dyproj is the *y*-axis diffusivity in the area of the projection fibers, and Dzassoc is the z-axis diffusivity in the area of the association fibers (Figure 3).

### Statistical Analysis

The SPSS version 21.0 software (Inc., Chicago, IL, United States) was used for the statistical analysis. NRS, VAS, SAS, and SDS scores, diffusivity, and the ALPS index are expressed as means ± standard deviations. Bland-Altman plot analysis was used to analyze the consistency of results between NRS and VAS pain assessment methods. Kruskal-wallis analysis was used to test for differences in diffusivity, ALPS index, MMSE, SAS, and SDS scores among the three groups. Wilcoxon rank-sum analysis was used to test for differences in diffusivity, and ALPS index in participants in the CP group from before to after pain treatment. Changes in SAS and SDS scores in the CP group before and after pain treatment were compared using paired-samples t-test. Chi-square tests were used to analyze differences in sex ratio, age composition, and Fazekas score among the three groups. Pearson’s correlation was used to analyze correlations between diffusivity and the ALPS index and age, Fazekas score, NRS score, VAS score, pain duration, SAS score, and SDS score. Statistical significance was set to *p* < 0.05.

## Results

### Demographic and Clinical Features

Because glymphatic system function can be influenced by age, sex, white matter lesions, and emotional state (Nedergaard and Goldman, 2020; Gertje et al., 2021), we firstly compared the age and sex compositions and brain white matter T2-hyperintensity among the CP, PLC and NC group. After analysis, no significant differences were found among groups in age (54.33 ± 7.28 years vs. 56.30 ± 6.31 years vs. 52.77 ± 10.34 years, *p* = 0.387), age composition (*p* = 0.069), or sex composition (*p* = 0.444). The Fazekas scores of the three groups were as follows: CP group: score of 0, 14 (35%); score of 1, 12 (30%); score of 2, 12 (30%); score of 3, 2 (5%). PLC group: score of 0, 9 (33.3%); score of 1, 9 (33.3%); score of 2, 9 (33.3%); score of 3, 0.NC group: score of 0, 9 (30%); score of 1, 13 (43.3%); score of 2, 7 (23.3%); score of 3, 1 (3.33%). No significant difference was found among groups for the Fazekas score (*p* = 0.876). SAS and SDS scores were both higher in the CP group than in the NC group (SAS: 58.98 ± 8.22 vs. 44.80 ± 3.55, *p* < 0.001; SDS: 58.80 ± 7.79 vs. 48.10 ± 2.66, *p* < 0.001). And no significant difference was found between CP group and PLC group for SAS and SDS scores, although CP group showed higher scores (*p* = 0.134). No significant difference was found among groups for MMSE score (*p* = 0.249).

### The Consistency of the Numeric Rating and Visual Analog Scales Scores

**FIGURE 4:**
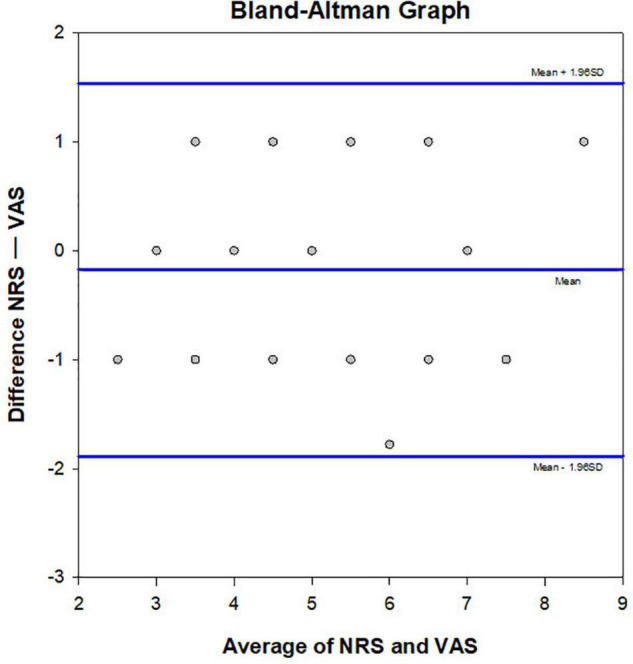
Bland Altman plot. Consistency of the NRS and VAS methods was verified (*n* = 40). The averages of the two scores were compared and plotted. The central line represents the mean difference, and the upper and lower lines indicate the 95% limits of agreement.

Results of Bland-Altman plot analysis showed that the two methods had high consistency in evaluating the degree of CP. The deviation value of the two methods was −0.175, the standard deviation was 0.873, the limits of agreement = −1.888, 1.5376, and the 95% bias confidence interval (CI) = −0.454, 0.104. The lower limit of agreement 95% CI = −2.371, −1.40, and the upper limit of agreement 95% CI = 1.054, 2.021 (Figure 4).

### Alterations in Diffusivity and Along the Perivascular Space Index in Cancer Pain

**FIGURE 5:**
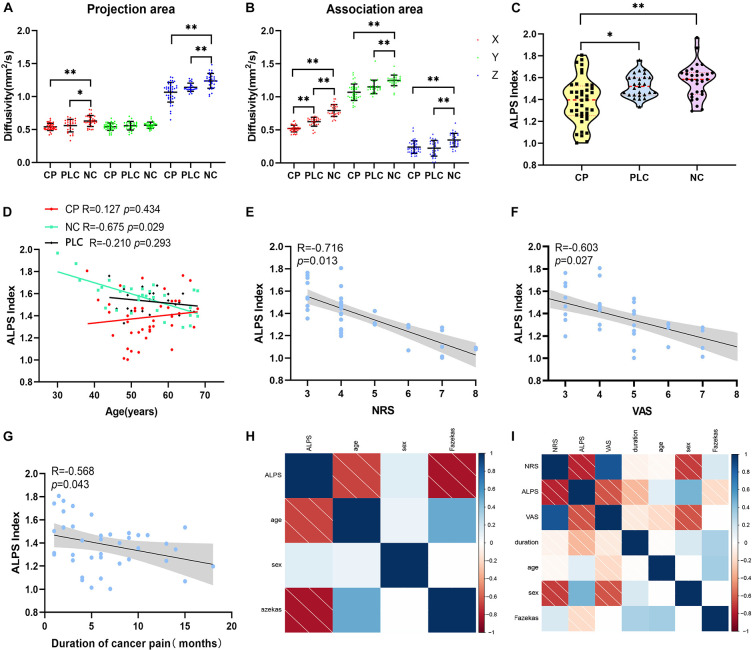
Differences in diffusivity and ALPS index among the CP, PLC, and NC groups. **(A,B)** Comparisons of Dx, Dz, Dy of the projection and association fibers among the CP, PLC and NC groups. Diffusivity in the CP group was lower than that in the NC group, except for Dyproj. And Dxassoc in the CP group was lower than that in the PLC group. **(C)** Violin plot showing the comparison of the ALPS index among the NC, PLC and CP groups. The ALPS index of the CP group was significantly lower than that in the PLC and NC groups. **(D)** Correlation between ALPS index and age in the NC (green), PLC (black) and CP groups (red). In the NC group, the ALPS index was negatively correlated with age. However, in the CP and PLC groups, there were no correlations between the ALPS index and age. **(E,F)** The ALPS index was negatively correlated with NRS and VAS scores in the CP group. **(G)** Negative correlation between the ALPS index and pain duration. **(H,I)** The correlation factor matrices of the NC and CP groups; darker colors where the two factors converge indicate higher correlations. *p < 0.05, **p < 0.01.

Compared with the NC group, Dxproj (0.541 ± 0.054 vs. 0.629 ± 0.083, *p* < 0.001), Dzproj (1.066 ± 0.150 vs. 1.238 ± 0.112, *p* < 0.001), Dxassoc (0.521 ± 0.051 vs. 0.793 ± 0.089, *p* < 0.001), Dyassoc (1.070 ± 0.124 vs. 1.249 ± 0.080, *p* < 0.001), and Dzassoc (0.239 ± 0.095 vs. 0.345 ± 0.103, *p* < 0.001) were significantly lower in the CP group. However, there was no difference in Dyproj between the NC and CP groups (0.543 ± 0.009 vs. 0.566 ± 0.009, *p* = 0.355). Compared with the PLC group, Dxassoc (0.521 ± 0.051 vs. 0.624 ± 0.067, *p* < 0.001) was significantly lower in the CP group (*p* < 0.001) (Figures 5A,B). No significant differences were found for Dxproj (0.541 ± 0.054 vs. 0.559 ± 0.095, *p* = 0.453), Dyproj (0.543 ± 0.009 vs. 0.558 ± 0.065, *p* = 0.859), Dzproj (1.066 ± 0.150 vs. 1.141 ± 0.063, *p* = 0.488), Dyassoc (1.070 ± 0.124 vs. 1.150 ± 0.102, *p* = 0.070), and Dzassoc (0.239 ± 0.095 vs. 0.222 ± 0.119, *p* = 0.999) between CP and PLC group. In the NC group, Dxproj (*r* = –0.698, *p* < 0.001) and Dzproj (*r* = −0.661, *p* < 0.001) were negatively correlated with the Fazekas score. In the CP group, Dxproj was negatively correlated with NRS and VAS scores (r_NRS_ = –0.463, *p*_NRS_ = 0.003, r_VAS_ = –0.586, *p*_VAS_ < 0.001) and Dzassoc was negatively correlated with pain duration (*r* = –0.360, *p* = 0.023) (Table 2 and Table 3).

**TABLE 2:** Correlation analysis of CP group.

	NRS		VAS		Pain duration		Age		Fazekas score		SDS		SAS	
Dxproj	r = −0.463	*p* = 0.003	r = −0.586	*p* < 0.001	*r* = 0.184	*p* = 0.255	*r* = 0.211	*p* = 0.192	*r* = −0.050	*p* = 0.761	*r* = 0.065	*p* = 0.692	r = −0.138	*p* = 0.396
Dyproj	r = −0.099	*p* = 0.542	r = −0.263	*p* = 0.101	*r* = 0.023	*p* = 0.886	*r* = −0.060	*p* = 0.713	*r* = 0.286	*p* = 0.073	*r* = 0.290	*p* = 0.069	r = −0.006	*p* = 0.971
Dzproj	r = −0.081	*p* = 0.617	r = −0.111	*p* = 0.494	*r* = −0.082	*p* = 0.616	*r* = −0.064	*p* = 0.696	*r* = −0.063	*p* = 0.701	*r* = 0.020	*p* = 0.902	r = −0.080	*p* = 0.623
Dxassoc	r = −0.407	*p* = 0.063	r = −0.349	*p* = 0.059	*r* = −0.360	*p* = 0.023	*r* = 0.201	*p* = 0.215	*r* = −0.052	*p* = 0.749	*r* = 0.090	*p* = 0.580	r = −0.222	*p* = 0.169
Dyassoc	r = 0.267	*p* = 0.096	r = 0.203	*p* = 0.210	*r* = −0.123	*p* = 0.449	*r* = −0.070	*p* = 0.666	*r* = −0.163	*p* = 0.314	*r* = 0.215	*p* = 0.182	r = 0.009	*p* = 0.956
Dzassoc	r = 0.562	*p* = 0.000	r = 0.462	*p* = 0.003	*r* = 0.242	*p* = 0.133	*r* = −0.197	*p* = 0.222	*r* = 0.040	*p* = 0.807	*r* = –0.020	*p* = 0.905	r = 0.259	*p* = 0.107
ALPS index	r = −0.876	*p* = 0.013	r = −0.793	*p* = 0.027	*r* = −0.322	*p* = 0.043	*r* = −0.230	*p* = 0.056	*r* = −0.190	*p* = 0.242	*r* = –0.036	*p* = 0.826	r = −0.261	*p* = 0.104

**TABLE 3:** Correlation analysis of PLC and NC groups.

	Age		Fazekas score		SDS		SAS	
**PLC group**								
Dxproj	r = −0.171	*p* = 0.394	*r* = 0.316	*p* = 0.109	r = 0.143	*p* = 0.814	*r* = 0.134	*p* = 0.444
Dyproj	r = 0.112	*p* = 0.577	*r* = 0.346	*p* = 0.587	r = 0.262	*p* = 0.612	*r* = 0.078	*p* = 0.873
Dzproj	r = 0.192	*p* = 0.336	*r* = 0.044	*p* = 0.827	*r* = 0.168	*p* = 0.604	*r* = −0.110	*p* = 0.661
Dxassoc	r = 0.461	*p* = 0.066	*r* = 0.063	*p* = 0.757	*r* = 0.304	*p* = 0.173	*r* = 0.236	*p* = 0.489
Dyassoc	r = 0.794	*p* = 0.207	*r* = 0.270	*p* = 0.173	*r* = 0.311	*p* = 0.114	*r* = 0.117	*p* = 0.542
Dzassoc	r = 0.095	*p* = 0.638	*r* = 0.437	*p* = 0.063	*r* = 0.226	*p* = 0.501	*r* = 0.007	*p* = 0.603
ALPS index	r = −0.210	*p* = 0.293	*r* = 0.447	*p* = 0.019	*r* = 0.141	*p* = 0.441	*r* = 0.163	*p* = 0.563
**NC group**								
Dxproj	*r* = –0.273	*p* = 0.144	*r* = −0.698	*p* < 0.001	*r* = 0.074	*p* = 0.697	*r* = −0.127	*p* = 0.503
Dyproj	*r* = –0.194	*p* = 0.304	*r* = −0.024	*p* = 0.900	*r* = −0.432	*p* = 0.064	*r* = −0.157	*p* = 0.407
Dzproj	*r* = –0.448	*p* = 0.060	*r* = −0.661	*p* < 0.001	*r* = −0.183	*p* = 0.332	*r* = −0.085	*p* = 0.654
Dxassoc	*r* = 0.693	*p* = 0.244	*r* = −0.366	*p* = 0.543	*r* = 0.042	*p* = 0.826	*r* = −0.143	*p* = 0.451
Dyassoc	*r* = 0.233	*p* = 0.216	*r* = −0.041	*p* = 0.829	*r* = 0.688	*p* = 0.248	*r* = 0.084	*p* = 0.661
Dzassoc	*r* = 0.627	*p* = 0.102	*r* = 0.304	*p* = 0.103	*r* = 0.253	*p* = 0.178	*r* = −0.044	*p* = 0.818
ALPS index	*r* = −0.675	*p* < 0.001	*r* = −0.826	*p* < 0.001	*r* = 0.045	*p* = 0.813	*r* = −0.319	*p* = 0.840

Results of the ALPS index analysis showed that the ALPS index in the CP group was significantly lower than that in the PLC and NC group (1.571 ± 0.153 vs. 1.526 ± 0.103 vs. 1.386 ± 0.207, *p* = 0.0002; Figure 5C). The ALPS index was negatively correlated with age and Fazekas score in the NC group (*r*_age_ = –0.675, *p*_age_ = 0.000; *r*_Fazekas_ = –0.626, *p*_Fazekas_ = 0.012), which is consistent with previous research (Figures 5D,H; McKnight et al., 2021). However, the ALPS index was not correlated with age or Fazekas score in the CP group (*r*_age_ = −0.230, *p*_age_ = 0.056; *r*_Fazekas_ = −0.190, *p*_Fazekas_ = 0.242; Figure 5I). The ALPS index in the CP group was negatively correlated with NRS and VAS scores (*r*_NRS_ = −0.716, *p*_NRS_ = 0.013; *r*_VAS_ = −0.603, *p*_VAS_ = 0.027; Figures 5E,F,I). In addition, we found that the ALPS index in the CP group was negatively correlated with pain duration (*r* = −0.568, *p* = 0.043; Figures 5G,I). Finally, we did not find any significant correlations between diffusivity in any x, y or z direction, ALPS index and emotional scores in either the CP or NC group (*p* > 0.05). Besides, no significant correlation was found in PLC group between diffusivity, ALPS index and other parameters (*p* > 0.05) (Tables 2, 3).

### Diffusivity and Along the Perivascular Space Index Changes in the Cancer Pain Group After Pain Intervention

**FIGURE 6:**
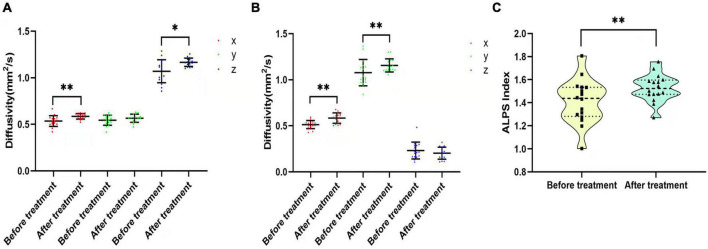
Diffusivity and ALPS index changes before and after pain intervention. **(A,B)** Comparisons of Dx, Dy, and Dz in the projection and association fibers between before and after pain treatment. Diffusivity increased after treatment, except for Dyproj and Dzassoc. **(C)** Violin plot shows the comparison of the ALPS index between before and after pain treatment. The ALPS index increased significantly after pain treatment. *p < 0.05, **p < 0.01.

In the CP group, there was no significant difference in diffusivity or ALPS index between participants who had been treated with drugs before recruitment and those who had not (*p* > 0.05). The 16 participants in the CP group who were enrolled in the follow-up group were treated with pain medication for 1 month. NRS, VAS, SAS, and SDS scores of these 16 participants decreased after the intervention (NRS: 4.50 ± 1.317 vs. 1.88 ± 0.619, *p* < 0.001; VAS: 4.38 ± 1.409 vs. 1.88 ± 0.696, *p* = 0.000; SDS: 59.64 ± 9.014 vs. 58.45 ± 7.258, *p* = 0.001; SAS: 59.73 ± 6.784 vs. 58.91 ± 7.409, *p* = 0.001). Dxproj (0.535 ± 0.057 vs. 0.586 ± 0.030, *p* = 0.002), Dxassoc (0.513 ± 0.044 vs. 0.584 ± 0.057, *p* < 0.001), Dzproj (1.070 ± 0.123 vs. 1.166 ± 0.045, *p* = 0.018), Dxassoc (0.513 ± 0.044 vs. 0.584 ± 0.057, *p* < 0.001), Dyassoc (1.077 ± 0.143 vs. 1.156 ± 0.071, *p* = 0.003), and ALPS index (1.412 ± 0.193 vs. 1.528 ± 0.119, *p* = 0.012) were significantly higher after the intervention (Figure 6). No significant alterations were found for Dyproj (0.544 ± 0.053 vs. 0.565 ± 0.044, *p* = 0.231) and Dzassoc (0.232 ± 0.091 vs. 0.204 ± 0.065, *p* = 0.489) after intervention. Although diffusivity and the ALPS index showed an increasing trend with decreases in NRS and VAS scores after pain intervention, there was no correlation between these measures (*p* > 0.05). Moreover, there were no significant correlations among diffusivity, ALPS index, and emotional scores after treatment (*p* > 0.05) (Table 4).

**TABLE 4:** Correlation analysis of diffusivity and ALPS index after pain intervention.

	NRS		VAS		SDS		SAS	
Dxproj	*r* = −0.103	*p* = 0.705	*r* = −0.105	*p* = 0.388	*r* = 0.140	*p* = 0.682	*r* = −0.061	*p* = 0.859
Dyproj	*r* = −0.056,	*p* = 0.837	*r* = 0.202	*p* = 0.453	*r* = 0.497	*p* = 0.120	*r* = 0.157	*p* = 0.644
Dzproj	*r* = 0.041	*p* = 0.880	*r* = −0.091	*p* = 0.737	*r* = 0.348	*p* = 0.294	*r* = 0.406	*p* = 0.215
Dxassoc	*r* = −0.010	*p* = 0.969	*r* = −0.084	*p* = 0.756	*r* = 0.382	*p* = 0.246	*r* = −0.025	*p* = 0.942
Dyassoc	*r* = 0.293	*p* = 0.272	*r* = 0.325	*p* = 0.220	*r* = 0.182	*p* = 0.591	r = 0.174	*p* = 0.610
Dzassoc	*r* = −0.017	*p* = 0.951	*r* = 0.009	*p* = 0.975	*r* = −0.225	*p* = 0.505	*r* = −0.320	*p* = 0.338
ALPS index	*r* = −0.008	*p* = 0.978	*r* = −0.288	*p* = 0.279	*r* = 0.278	*p* = 0.408	*r* = 0.226	*p* = 0.504

## Discussion

In this study, DTI-ALPS was used as a non-invasive method to detect the alteration of glymphatic function in CP caused by bone metastasis, which is the most common forms of persistent and severe CP (Hong et al., 2020; Yoneda et al., 2021). We firstly found the alteration of glymphatic function under CP. Furthermore, the relationship between glymphatic function and CP was revealed. Our findings are helpful to further understand the functional characteristics of brain under cancer pain, and also has important hints for the evaluation of cancer pain through brain function detection.

To date, although there are various methods to treat CP, such as three-stage analgesics, nerve blocks, and Chinese acupuncture point stimulation, 50% of patients continue to experience CP. In addition, some patients experience side effects, such as increased drug tolerance or even addiction due to drug abuse during the treatment process; this is because the CP experience is subjective, with no objective indicator for CP evaluation in clinical pain management (Gallagher et al., 2017; Neufeld et al., 2017). Therefore, an objective biomarker that reflects the status of CP will help to accurately quantify CP in the clinic and will be highly valuable for the formulation of effective treatment plans for CP.

In chronic pain stimulation, numerous microstructural and functional changes occur in the brain, such as synaptic plasticity, blood perfusion, and connectivity properties of the functional and structural networks (Kuner and Flor, 2017; Iwabuchi et al., 2020; Barroso et al., 2021). These changes are closely related to the onset of pain, which may not only be adaptive changes in response to peripheral pain stimulation but also a key intermediate link to pain perception (Prinsloo et al., 2014). Han et al. found using tracer-based MRI that peripheral pain stimulation induces changes in ISF drainage and the spatial structure of ECS in the deep brain (Li et al., 2020). Excitation of neurons decreased the volume fraction (α) of the ECS in the deep brain and the clearance rate of ISF toward the superficial cortex (Li et al., 2020).

In our study, we found that diffusivity of the CP group decreased in the primary directions of the projection and association fibers and perivascular space was significantly lower than that in the NC group; moreover, the ALPS index in the CP group was also significantly lower than that in the PLC and NC group, which indicated that ISF drainage in both the brain parenchyma and perivascular space was impaired. We speculate that these drainage alterations were caused by the following mechanisms: (1) in our previous research, we found that myelinated fiber tracts regulated ISF drainage in the deep brain, and the ISF drained into the superficial cortex along the myelinated fiber tracts, which constitutes the upstream structure of the glymphatic system in the cortex (Wang et al., 2019). As previously reported by Han et al., peripheral pain stimulation leads to morphological changes of brain cells, which results in spatial structure changes of the ECS in the deep brain, obstructing ISF drainage into the cortex and affecting the function of the glymphatic system downstream. This results in a decrease in diffusivity, which reflects the drainage directions of the myelinated fiber tracts and perivascular space (Li et al., 2020). (2) The release of neurotransmitters, such as norepinephrine, increases under chronic pain stimulation, which may cause arteries in the brain to contract and decrease pulsation, affecting the function of the glymphatic system (Xie et al., 2013; IsHak et al., 2018; Goldman et al., 2020). Norepinephrine promotes the activation of microglia, which interact with other brain cells, such as neurons and astrocytes, via ‘crosstalk,’ causing changes in the activity and metabolism of cells, further affecting the function of the glymphatic system (Malcangio, 2019). (3) Because chronic pain is a risk factor for depression and neurodegenerative diseases, such as AD, pain stimulation may also affect brain waste clearance and the drainage function of perivascular spaces (IsHak et al., 2018). All of these mechanisms can influence the overall ISF drainage in the brain.

Moreover, we found that the ALPS index was correlated with age and Fazekas score in the NC group, which is consistent with previous studies (McKnight et al., 2021). However, this was not observed in the CP group, which showed a negative correlation between the ALPS index and degree of pain, where the higher the level of pain, the lower the ALPS index. Following pain intervention, the ALPS index increased with pain relief, which suggested that the glymphatic system is mainly affected by pain stimulation under CP, and the ALPS index is a parameter that reflects the function of the glymphatic system and has a certain indication function on CP. Additionally, the ALPS index in the CP group was negatively correlated with the duration of pain, which suggested that the longer the pain duration, the more serious the impairment to the glymphatic system. However, whether there is an interaction between the degree of pain and pain duration on drainage function remains to be elucidated in a larger sample. It is worth noting that there was no correlation between the ALPS index and degree of pain after pain intervention. We speculate that this is because the low degree of pain before the intervention in the participants who were enrolled in the follow-up, and their pain was effectively controlled after intervention, and the change in the ALPS index of them was not significant.

Our study has the following shortcomings. Firstly, the method we applied only describes ISF drainage in the paravalvular space at the level of the lateral ventricle via a mathematical formula; thus, it lacks intuition compared with that of the tracer-based method. Moreover, ISF drainage in the brain may be regionalized (Wang et al., 2019; Harrison et al., 2020), which means that the functions and spatial structures may be different among difference brain regions, and the measurement of drainage function for one region may not reflect the functional alteration of the whole brain. Secondly, a larger sample is needed to investigate influencing factors, such as analgetic dosage, of glymphatic system changes in patients with CP.

In conclusion, by applying DTI-ALPS, we found a correlation between glymphatic system function and CP and that the ALPS index may have potential value for evaluating CP. In the future, the ALPS index, combined with biological indicators, such as gene polymorphisms, may allow more objective and individualized CP assessments. Furthermore, the association between the glymphatic system and pain stimulation may help in the development of non-pharmaceutical interventions for pain, where pain may be alleviated via the regulation of glymphatic function (Goldman et al., 2020).

## Data Availability Statement

The original contributions presented in the study are included in the article/supplementary material, further inquiries can be directed to the corresponding author/s.

## Ethics Statement

The studies involving human participants were reviewed and approved by Ethics Committee of Tianjin Medical University Cancer Institute and Hospital. The patients/participants provided their written informed consent to participate in this study. Written informed consent was obtained from the individual(s) for the publication of any potentially identifiable images or data included in this article.

## Author Contributions

AW and ZY designed the study. LC collected the patients. XY collected the images. AW and LY processed the date and images. CT, MZ, XW, and YZ analyzed date and contributed to the original draft. AW, CT, and ZY reviewed and edited the manuscript. All authors contributed to the article and approved to the submitted version.

## Conflict of Interest

LY was employed by Zhejiang MedicalTech Therapeutics Company Co., Ltd. The remaining authors declare that the research was conducted in the absence of any commercial or financial relationships that could be construed as a potential conflict of interest.

## Publisher’s Note

All claims expressed in this article are solely those of the authors and do not necessarily represent those of their affiliated organizations, or those of the publisher, the editors and the reviewers. Any product that may be evaluated in this article, or claim that may be made by its manufacturer, is not guaranteed or endorsed by the publisher.

## Footnotes

1
https://fsl.fmrib.ox.ac.uk/fsl/fslwiki/
